# False negative rates in *Drosophila *cell-based RNAi screens: a case study

**DOI:** 10.1186/1471-2164-12-50

**Published:** 2011-01-20

**Authors:** Matthew Booker, Anastasia A Samsonova, Young Kwon, Ian Flockhart, Stephanie E Mohr, Norbert Perrimon

**Affiliations:** 1 Department of Genetics, Harvard Medical School, (77 Avenue Louis Pasteur), Boston, Massachusetts, (02115), USA; 2 Howard Hughes Medical Institute, (77 Avenue Louis Pasteur), Boston, Massachusetts, (02115), USA; 3 Department of Molecular Biology, Cell Biology, and Biochemistry, Brown University, (185 Meeting Street), Providence, Rhode Island, (02192), USA

## Abstract

**Background:**

High-throughput screening using RNAi is a powerful gene discovery method but is often complicated by false positive and false negative results. Whereas false positive results associated with RNAi reagents has been a matter of extensive study, the issue of false negatives has received less attention.

**Results:**

We performed a meta-analysis of several genome-wide, cell-based *Drosophila *RNAi screens, together with a more focused RNAi screen, and conclude that the rate of false negative results is at least 8%. Further, we demonstrate how knowledge of the cell transcriptome can be used to resolve ambiguous results and how the number of false negative results can be reduced by using multiple, independently-tested RNAi reagents per gene.

**Conclusions:**

RNAi reagents that target the same gene do not always yield consistent results due to false positives and weak or ineffective reagents. False positive results can be partially minimized by filtering with transcriptome data. RNAi libraries with multiple reagents per gene also reduce false positive and false negative outcomes when inconsistent results are disambiguated carefully.

## Background

The success of RNAi high throughput screening (HTS) relies on low experimental rates of false negative and false positive results, which in turn depend on the efficacy and specificity of the RNAi reagents, respectively (reviewed in [1,2]). False positive results can arise from at least the following causes: experimental noise inherent to large-scale studies, bias associated with a particular screen assay, incorrect gene models, and arguably most importantly, reagent-specific off-target effects (OTEs) (reviewed in [3]). Similarly, false negative results can arise as the result of experimental noise [4,5], aspects of screen assay design, and incorrect gene models, protein stability, gene redundancy, but most importantly, the rate of false negative results depends on the efficacy of the RNAi reagents used in the screen.

The issue of false positive results associated with RNAi reagents has been a matter of extensive study in recent years for screens in both *Drosophila *and mammalian cells [6-11]. In *Drosophila *cell-based RNAi screens, the focus of this study, cultured cells are treated with long double-stranded RNAs (dsRNAs) as the reagent for knockdown. Sequence-associated false positive results have been observed and characterized to a significant extent [10,11]; however, the full cause of the phenomenon remains to be elucidated. There are a number of ways to identify false positives in a screen, for example using 'gold standard' rescue methods [12,13]. By contrast, the identification of false negatives is not as straightforward, as identification of a false negative result requires previous knowledge that a gene is involved in the process under analysis. Thus, rates of false negative results have been estimated for screens that investigated well-characterized pathways. For example, in a screen for Hedgehog (Hh) signaling factors, only nine of fourteen known components of the pathway were identified [14] and only seven of these passed additional validation [15], suggesting a rate of false negative results of nearly 48%. Similarly, in a screen for Wingless (Wg)/Wnt signaling, only 16 of 21 canonical components expressed in the cell line used were identified in the screen [16]. Interestingly, when the "hits" (positive results) from the Wg screen were re-tested using three independent dsRNAs, 70 of 204 genes tested scored with three independent dsRNAs but 68 scored with only two out of three, suggesting a false negative rate of 16% [15]. Altogether, these analyses have suggested that false negative rates may be in the order of 16% to 50% in RNAi HTS.

One caveat to the studies that to date have looked at false negative rates in RNAi HTS is that the sample sizes were small. In order to get a more global view of false negative rates in *Drosophila *cell-based RNAi HTS, we decided to perform a number of analyses on a larger set of screens. The data sets we analyzed were from RNAi screens performed at the *Drosophila *RNAi Screening Center (DRSC) [17] where a standardized screening platform enables both local and visiting scientists to perform high-throughput screens with dsRNAs in *Drosophila *cell tissue culture. Each of the screens we analyzed used essentially the same dsRNA library (DRSC "2.0") and a standard cell line (S2, S2R+ or Kc167), such that variability due to equipment and reagents should be minimal. We also used data from DRSC screens in conjunction with an analysis of the transcriptome of cell lines [18] to estimate an overall false positive rate among long dsRNAs of roughly 1% and a false negative rate due to ineffective or weak dsRNAs of at least 8%. Furthermore, we find that the presence of multiple RNAi reagents per gene in a screening library can be a statistically powerful means of reducing false positive and negative results, although careful consideration must be made regarding the disambiguation of inconsistent results obtained with multiple reagents directed against the same target gene.

## Results

### Estimation of false negative rates using data from RNAi reagents directed against ribosome and proteasome components

The proteasome and ribosome are two well-characterized complexes in the cell that perform the essential functions of protein degradation and protein assembly, respectively. Because of the broad functionality of the ribosome and proteasome in basic cell metabolism, we reasoned that dsRNAs targeting components of these complexes might affect the output of a wide range of RNAi screens. Indeed, we find that dsRNAs targeting ribosomal or proteasome components frequently score as "hits" (positive results) in many screens thus making them particularly useful for analysis of false negative results. We used the Gene Ontology (GO) annotations at FlyBase [19] to select 185 genes with the GO:0005840: Ribosome annotation, and 58 genes with GO:0000502: Proteasome Complex (Additional file 1). Of the 185 ribosomal genes, a sub-set of 94 genes are also annotated with GO:0022626: Cytosolic Ribosome.

**Figure 1:**
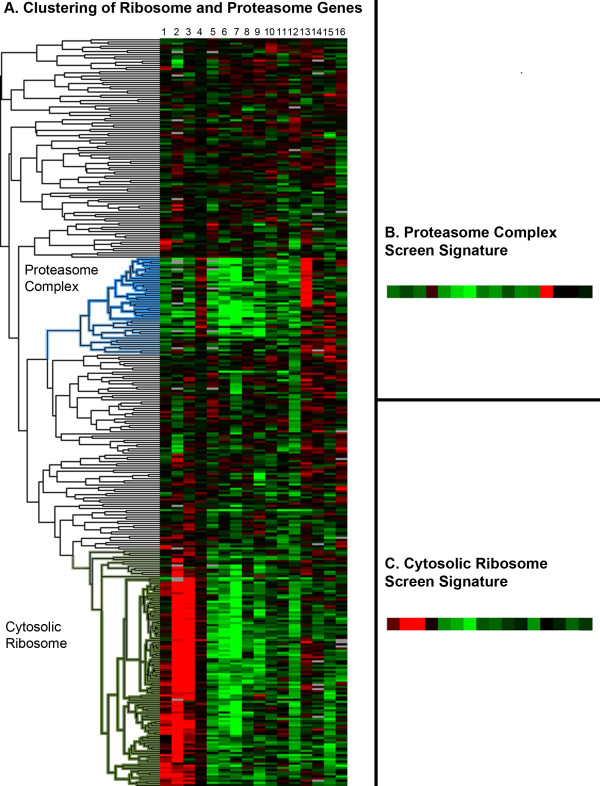
**Identification of Proteosome and Ribosome signatures in RNAi screens**. All dsRNAs included in the dendrogram target 243 proteasome and ribosome-related genes. Red indicates an increase in signal and green indicates a decrease in signal. (A) Results of clustering RNAi phenotypes in 16 screens of dsRNAs targeting ribosome and proteasome genes as defined by GO terms (see Materials and Methods). The proteasome complex and cytosolic ribosome clusters are highlighted in blue and green, respectively. The simple majority of dsRNAs outside these two clusters target mitochondrial ribosome components. (B) Consensus screen signature of the proteasome complex cluster. Each small square represents the mean Z-score of the dsRNAs in the proteasome complex cluster across a single screen. (C) Consensus screen signature of the cytosolic ribosome cluster. The 16 screens are as follows from the left to the right: 1. Hormone receptor screen, plate-reader (unpublished), 2. Oncogenesis screen, plate-reader (unpublished), 3. Protein degradation screen, plate-reader (unpublished), 4. RNA processing screen, plate-reader (unpublished), 5. Mitochondrial calcium ion and proton antiporter screen, plate-reader [37], 6. Toxicity screen, plate-reader (unpublished), 7. Dengue virus host factors screen, image-based [38], 8. Ion homeostasis screen, plate-reader (unpublished). 9. Pathogen infection screen, image-based (unpublished), 10. Signaling pathway screen, plate-reader (unpublished), 11. Ion transport screen, plate-reader (unpublished), 12. Cytoskeleton regulation screen, image-based (unpublished), 13. Chromatin regulation screen, image-based (unpublished), 14. *Francisella tularensis *infection screen, plate-reader [39], 15. mRNA processing screen, plate-reader (unpublished), 16. Protein secretion screen, plate-reader (unpublished).

We next selected 16 screens performed at the DRSC (see Materials and Methods) using version 2 of the DRSC genome-wide library, which was designed to minimize OTEs [15], and determined the scoring pattern of the ribosome and proteasome set for these screens. Two prominent clusters with strongly scoring dsRNAs clearly emerge (Figure 1A; Additional file 1): a "cytosolic ribosome cluster" that consists of 79 genes enriched for GO:0022626: Cytosolic Ribosome and a "proteasome complex cluster" that consists of 36 genes enriched for GO:0000502: Proteasome Complex. For each cluster, a "screen signature" was calculated by determining for each screen the mean Z-score of the dsRNAs in the cluster. The screen signatures for the proteasome complex and cytosolic ribosome clusters are shown in Figures 1B and 1C, respectively. Outside of these two clusters, the majority of dsRNAs are those that target components of mitochondrial ribosome. Unlike the cytosolic ribosome components, these do not appear to show strong phenotypes across multiple RNAi screens.

Some cytosolic ribosome and proteasome complex genes are absent from their respective clusters and lack functionally typical screen signatures. Overall, 22 of 94 cytosolic ribosome genes and 29 of 58 proteasome complex genes failed to yield the appropriate screen signature (Additional file 2). Possibilities for these failures include functional mis-annotation of genes or protection from loss-of-function phenotypes due to gene redundancy. Additionally, some of the non-clustering cytosolic ribosome and proteasome complex genes may represent false negatives due to insufficient knockdown.

**Figure 2:**
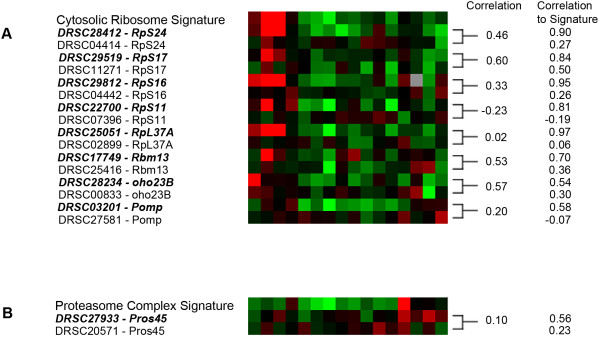
**Estimation of the rate of false negatives for the Ribosome (A) and Proteasome (B) set**. Red indicates an increase in signal and green indicates a decrease in signal. (A) The cytosolic ribosome screen signature is compared to the screen signatures in those cases where one dsRNA is part of the cytosolic ribosome cluster and the other is not. dsRNAs with a screen signature similar to the consensus cytosolic ribosome signature are italicized. Pearson's correlation is shown between dsRNAs that target the same gene as well as the correlation between each dsRNA and the consensus signature. (B) Similar comparison for the proteasome complex screen signature.

When there is only a single dsRNA targeting the gene that did not result in the predicted screen signature, it is not possible to distinguish among potential causes of negative results. Fortunately, however, DRSC library version 2 has two or more dsRNAs per gene for many genes represented in the library. In principle, because dsRNAs that target the same gene should yield similar screen signatures, we can ask if this is the case when two such dsRNAs against the same gene exist in the collection. Within the proteasome complex and cytosolic ribosome clusters, there are 51 genes represented by two dsRNAs in the dsRNA library for a total of 103 dsRNAs (in one case three dsRNAs targeted a single gene). Of these 51 genes, 42 have dsRNAs that exhibit the appropriate screen signatures. In 9 cases however, only one dsRNA appears either in the cytosolic ribosome cluster (Figure 2A) or the proteasome complex cluster (Figure 2B). In 8 of these 9 cases, the gene target is well known and functionally consistent with the cluster in which one of its dsRNAs appears (DRSC03201, which targets *Pomp*, clusters in the periphery of the cytosolic ribosome cluster and is most likely a false positive). Because for 8 genes, one dsRNA gave the expected screen signature but the other did not, the non-signature dsRNAs are likely false negatives. Thus, we conclude that 8 out of 103 dsRNAs failed to cluster as expected, yielding to a false negative rate due to ineffective RNAi reagents at 8%. Because this estimate is derived from a meta-analysis of multiple screens, the most likely explanation for these false negative is weak or ineffective dsRNAs rather than statistical noise from an individual screen.

Note that the 8% rate is likely under-estimated since we did not take into account the false negative rate present in the initial screen. Our reasoning for not including them is that we do not know whether the genes that did not score initially should have scored in the assays. Regardless, if we do include those, the false negative rate is higher and reaches 34% [(22+29)/(94+58)]. Test of additional dsRNAs will be necessary to address whether these are genuine false negatives or not.

### Use of focused RNAi libraries with multiple reagents per gene as a strategy to minimize the rate of false negative results

The past and current DRSC genome-wide *Drosophila *libraries included redundant dsRNAs for only a subset of the genome, thus limiting our ability to fully assess rates of false negative results using full-genome screen datasets. To address this issue, we generated sub-libraries containing multiple RNAi reagents (2 to 4) for several specific gene families (see Materials and Methods), such that analysis of results from a sub-library should supplement the results reported from genome-wide screens. Similar to the cluster analysis presented above, the sub-library sets allow for comparison of the behavior of multiple reagents per gene. Additionally, the layout of the sub-library assay plates was designed with an outer perimeter of wells that lack dsRNAs to reduce the possible influence edge effects that occur in many screens [20]. Currently, four sub-libraries have been generated: a kinases and phosphatases sub-library (K/P), a transcription factor and DNA binding sub-library (TRXN), a transmembrane domain-containing protein sub-library (TM), and a library which covers genes involved in ubiquitination and related processes (UBIQ) (Table 1). Like version 2 of the DRSC genome-wide library, these sub-libraries were designed with SnapDragon [21] to avoid sequences known to cause OTEs (see Materials and Methods).

**Table 1:** List of RNAi sub-libraries.

Library	Gene Set	Number of Genes	dsRNAs per Gene
DRSC K/P	Kinases & Phosphatases	563	2-4

DRSC TRXN	Transcription Factors	993	2

NYU-DRSC UBIQ	Ubiquitin-Related Genes	439	2-3

NYU-DRSC TM	Transmembrane Proteins	1729	2

The gene lists are available [40]. The UBIQ and TM sub-libraries were developed in conjunction with the RNAi Core Facility at New York University (NYU) [41].

### K/P screen for JAK/STAT signaling pathway components: a case study in identification of false discovery rates

To demonstrate the utility of focused libraries with multiple amplicons per gene, we screened the K/P set for factors involved in the JAK/STAT pathway. S2R+ cells were transfected with dsRNA and both 10xSTAT-firefly luciferase and actin-renilla luciferase constructs as previously described ([22]; Materials and Methods). The pathway was stimulated three days later by the addition of S2NP cells transfected with a plasmid expressing the Unpaired ligand [22], and JAK/STAT pathway activity quantified by measuring firefly luciferase activity. Renilla luciferase activity was used for normalization. The redundant coverage of genes in the K/P library provides an opportunity to compare the behavior of dsRNAs that target the same gene. The K/P set contains two canonical positive regulators of JAK/STAT signaling with three dsRNAs each: *domeless *(*dome*), which was initially annotated as a phosphatase [23], and the kinase *hopscotch *(*hop*) (reviewed in [24]). All dsRNAs targeting *dome *and *hop *were strong hits in the screen, with Z-scores less than -4. The K/P set also contains one canonical negative regulator of JAK/STAT signaling, *Ptp61F*. The three dsRNAs for this gene did not score, most likely because over-stimulation with the act-upd construct makes it difficult to detect negative regulators of JAK/STAT signaling in S2R+ cells [25].

**Figure 3:**
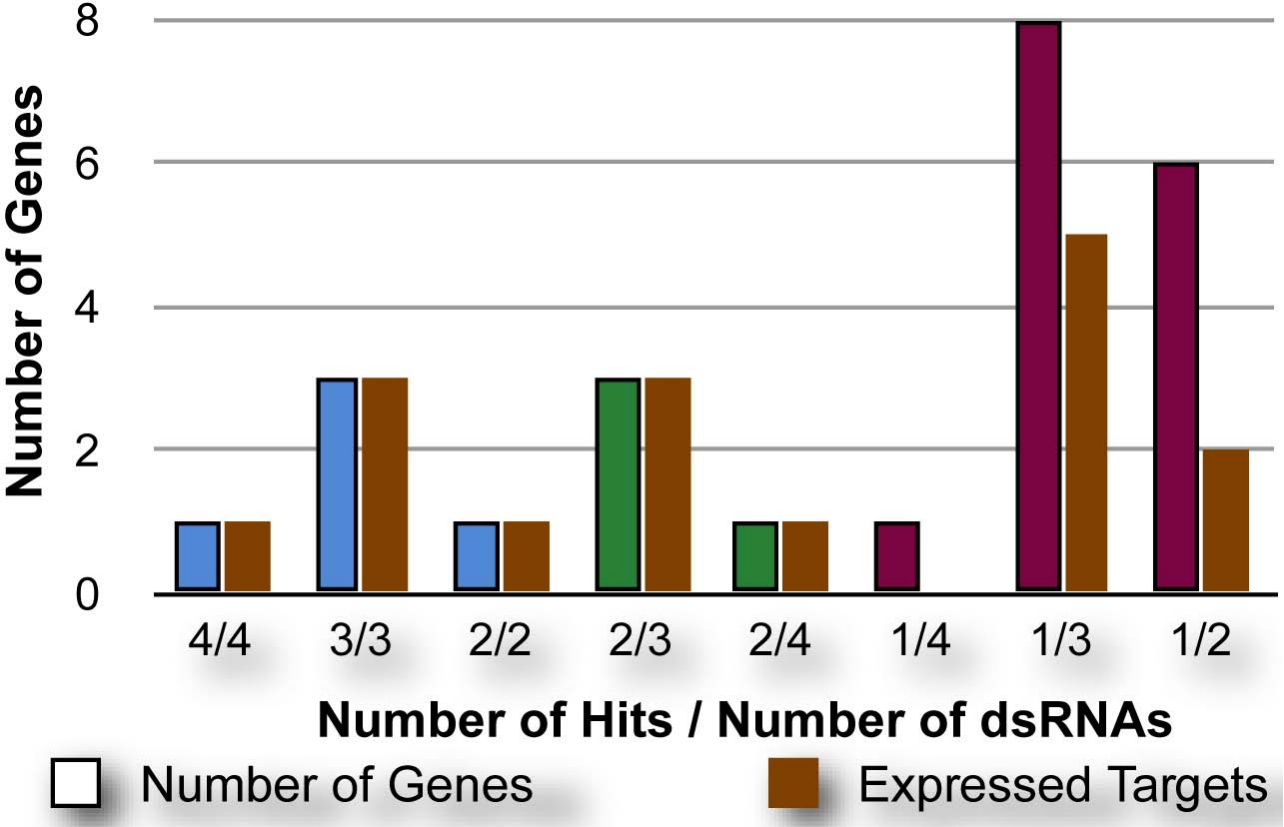
**Results of the JAK/STAT signaling screen**. The number of genes binned by the number of dsRNAs that scored out of the number of dsRNAs screened is shown. These are color-coded further: Blue for category 1 in which all dsRNAs scored, Green for category 2 in which at least two dsRNAs scored and maroon for category 3 in which only one dsRNA scored. The beige column to the right indicates the number of genes in each binned category that are expressed in S2R+ cells.

For further analysis, we selected those dsRNAs with Z-scores with an absolute value of 2 or greater across both replicates, which in this case included dsRNAs targeting 24 genes (Figure 3, Table 2). We then compared these to the Z-scores of the other dsRNAs in the K/P set that target the same gene and transcripts. In some cases, the scores obtained with all dsRNAs directed against a particular gene were consistent, whereas in other cases, some dsRNAs directed against a single gene were phenotypically inconsistent. We categorized the results of dsRNAs into three categories: In category 1, all dsRNAs directed against a given gene were hits. In category 2, at least 2 dsRNAs were hits but there was at least one which did not score significantly. In category 3, only 1 dsRNA directed against a gene yielded a significant result. Out of 24 genes, 5 had positive results for all dsRNAs (category 1), 4 were in category 2, and 15 were in category 3 (Figure 3, Table 2).

**Table 2:** Hits organized by genes in the K/P JAK/STAT screen.

Gene	Number of dsRNAs	Number of scoring dsRNAs	Number of non-scoring dsRNAs	Category	Expressed in S2R+ Cells
Abl	4	4	0	1	Yes

CycA	3	3	0	1	Yes

dome	3	3	0	1	Yes

hop	3	3	0	1	Yes

mts	2	2	0	1	Yes

CycE	3	2	1	2	Yes

Pp4-19C	4	2	2	2	Yes

CG17090	3	2	1	2	Yes

puc	3	2	1	2	Yes

CG34318 | CG8179	4	1	3	3	No

CanA1	3	1	2	3	No

CG4839	3	1	2	3	No

CG7597	3	1	2	3	Yes

CG9389	3	1	2	3	No

mtm	3	1	2	3	Yes

Pi3K21B	3	1	2	3	Yes

smi35A	3	1	2	3	Yes

Src42A	3	1	2	3	Yes

CG8509	2	1	1	3	No

gskt	2	1	1	3	No

htl	2	1	1	3	No

Myt1	2	1	1	3	Yes

Pp1-Y2	2	1	1	3	No

S6k	2	1	1	3	Yes

Genes were included on this list if at least one dsRNA yielded a Z-score of +/- 2 or better across both replicates. The number of dsRNAs in the K/P set is indicated as well as the number of those dsRNAs that were scored in the screen with a Z-score with an absolute value of 1.5 or better. Genes were binned into 3 categories: Category 1 contains genes where 4 out of 4 or 3 out of 3 or 2 out of 2 dsRNAs scored. Category 2 contains genes where 2 out of 3 or 2 out of 4 or 3 out of 4 dsRNAs scored. Category 3 contains genes where only 1 dsRNA scored. A gene was defined to be expressed in S2R+ if the FPKM value was greater than 1.

### Using transcriptome analysis to preferentially filter false positives

In those cases where we observed discrepancies (categories 2 and 3), we determined whether the targeted gene was expressed in S2R+ cells using expression datasets [18]. In principle, this information could be extremely useful for data curation, as dsRNAs that score but for which there is no evidence that the gene is expressed in the cell line tested are likely false positives. Importantly, transcriptome information may not only help to resolve many ambiguous false positive cases but also help identify false negatives, as the inconsistent dsRNAs that have been ruled out to be due to false positives should be enriched for false negatives.

**Figure 4:**
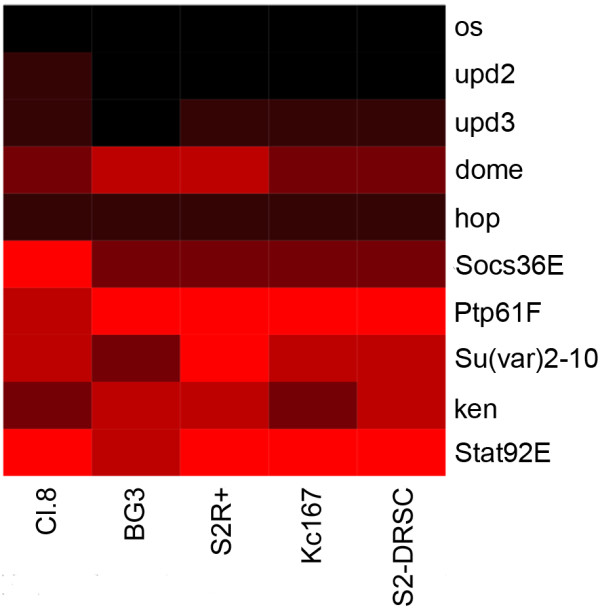
**Transformed expression levels of core components of JAK-STAT signaling pathway**. Genes expressed at low and high levels are displayed in gradations of black and red, correspondingly.

Analysis of the transcriptional activity in S2R+ cultured cells provides evidence for expression of 7,069 genes (see Materials and Methods). Of these, 6,223 (or 45%) of annotated protein-coding genes are expressed at elevated levels (FPKM >= 5). Of the genes in the K/P sub-library, 70% are expressed in S2R+ cells. Importantly, we found evidence that all of the core components of the JAK/STAT pathway required for signal transduction are expressed in S2R+ cells (Figure 4). Interestingly, the Upd ligands are either not expressed or expressed at low levels, suggesting that the JAK/STAT pathway is either not active or active at low levels in cultured cell lines, which is consistent with the fact that stimulation with act-upd was necessary to activate the pathway for our RNAi screen (see Materials and Methods).

Of the 24 genes found in the K/P screen, 16 are expressed in S2R+ cells (Figure 3, Table 2). All category 1 and category 2 genes are expressed and are represented by multiple scoring dsRNAs, suggesting that the few dsRNAs that did not score are most likely false negatives. Categories 1 and 2 represent results from 37 dsRNAs of which 5 did not score. Therefore, we estimate a false negative rate of ~13%, which is roughly consistent with the ~8% estimate from the ribosome and proteosome cluster analysis described above. All 8 of the unexpressed genes are limited to the 15 category 3 genes for which only a single dsRNA scored (Figure 3, Table 2). Therefore, these 8 genes should be considered false positive results and should be viewed as low priority for selection for additional validation.

Since only 7 of the 15 category 3 genes are expressed (47%), category 3 genes show no enrichment for expressed genes. This suggests that few, if any of category 3 genes, for which a single dsRNA scored, represent true positives. Thus, assuming that all 15 category 3 dsRNAs are false positives, the overall rate of false positives for this K/P screen is 1% since we screened 1,545 dsRNAs in total. It is important to note that although 1% appears to be an acceptable low rate, when the same false positive rate is shown as a percentage of the genes identified as positives in the screen, the figure is 62% (15 out of 24; Figure 3, Table 2), thus, underscoring the need for further validation of primary hit lists.

**Figure 5:**
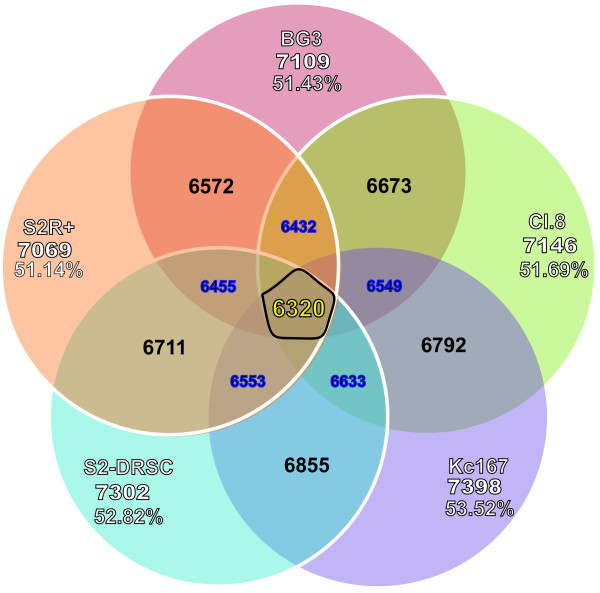
**Number of genes expressed in different cell lines at FPKM levels greater than one**. The cell lines included in the analysis are Kc167, Clone8, S2, BG3, and S2R+. 6,320 genes are expressed in all five cell lines.

Knowledge of the transcriptome of the cell line used in our K/P JAK/STAT screen allowed us to estimate the false positive rate, as few unexpressed genes are expected to be legitimate hits. Likewise, in any screen, failure to uncover some expected hits can sometimes be explained by the finding that those genes are simply not expressed in the specific cell line tested. In turn, this allows an estimate of false negatives in conjunction with multiple reagents per gene. To assist such analyses, we have analyzed gene expression based on deep-sequencing data obtained by the modENCODE consortium [18] for five *Drosophila *cell lines commonly used in RNAi HTS (Figure 5) and have made this data available on a website ([26], see Materials and Methods). Each of the cell lines expresses about 53% of protein-coding genes in the genome but the specific sub-set of genes that are expressed differs somewhat among the cell lines. We identified 6,230 genes expressed in all five cell lines, representing 46% of annotated protein-coding genes in release 5.22 of the *Drosophila *genome. False positives and false negatives can also potentially be filtered using tools based on protein interaction networks such as RNAiCut [27] and NePhe [28].

## Discussion

This study has focused on false negative rates among long dsRNAs used in *Drosophila *RNAi screens in cultured cells. Although the exact rates will vary depending on the reagent library, assay design, and the level of statistical noise, our analysis provides a detailed example of the issues that need to be considered carefully in the data analysis of an RNAi screen. Importantly, other RNAi reagents, such as siRNAs, shRNAs, and siRNA pools used in mammalian RNAi screens, have their own false positive and false negative rates and these are not necessarily the same as what we observed with *Drosophila *long dsRNAs. Regardless of the reagent used, however, any false negative rate significantly above zero will cause genes to be missed in an RNAi screen. Likewise, as shown in the K/P screen, even a very low false positive rate among the set of reagents can yield a very high proportion of false positives when expressed as a percentage of the hits obtained in an individual screen. Finally, our study illustrates how transcriptome data from the cell lines can be included as part of the data analysis to eliminate false positives.

The existence of false negatives due to ineffective RNAi reagents necessitates strategies for reducing their effects on the outcomes of RNAi screens. One obvious approach to minimize false negatives in screens is to use multiple, independently screened reagents per gene, as done in some recent RNAi screens [29,30]. In principle, use of multiple reagents per gene should reduce the number of false negatives, as a single ineffective RNAi reagent would be compensated by those that are effective. An obvious caveat to this, however, is that simply by including more reagents, the number of false positive results will also increase.

To explore how multiple RNAi reagents per gene could affect the outcome of a screen and to determine the best strategy for disambiguating results when different reagents yield inconsistent results, we devised a simple model of one, two, and three reagents per gene (Table 3). Furthermore, we examined three simple generalized disambiguation approaches and modeled how these approaches would affect the outcome of a screen. These disambiguation approaches are as follows: a lenient approach wherein a gene is considered a hit if any RNAi reagent directed against that gene scores above some threshold (Table 3, Rule A); a stringent approach that requires all reagents directed against the same gene to score (Table 3, Rule B); and an intermediate approach that requires more than half of the reagents directed against the same gene to score (Table 3, Rule C). For the purpose of this model, an RNAi "mini-pool" of reagents, such as is sometimes used for mammalian siRNA knockdown, or combinatorial knockdown with multiple dsRNAs, counts as a single RNAi reagent unless the individual components are tested separately.

**Table 3:** Model of RNAi reagent disambiguation methods under one, two or three reagents per gene.

	1 RNAi Reagent/Gene	2 RNAi Reagents/Gene	3 RNAi Reagents/Gene
"Lenient" Rule A: Number of False Negatives	*R_FN _*× *H*	$R_{FN}^{2}\times H$	$R_{FN}^{3}\times H$

"Lenient" Rule A: Number of False Positives	*R_FP _*× *N*	$\left(R_{FP}^{}\times 2-R_{FP}^{2}\right)\times N$	$\left(R_{FP}^{}\times 3-R_{FP}^{2}\times 3+R_{FP}^{3}\right)\times N$

"Stringent" Rule B: Number of False Negatives	*R_FN _*× *H*	$\left(R_{FN}\times 2-R_{FN}^{2}\right)\times H$	$\left(R_{FN}\times 3-R_{FN}^{2}\times 3+R_{FN}^{3}\right)\times H$

"Stringent" Rule B: Number of False Positives	*R_FP _*× *N*	$R_{FP}^{2}\times N$	$R_{FP}^{3}\times N$

"Balanced" Rule C: Number of False Negatives	*R_FN _*× *H*	$\left(R_{FN}\times 2-R_{FN}^{2}\right)\times H$	$\left(R_{FN}^{2}\times 3-R_{FN}^{3}\times 2\right)\times H$

"Balanced" Rule C: Number of False Positives	*R_FP _*× *N*	$R_{FP}^{2}\times N$	$\left(R_{FP}^{2}\times 3-R_{FP}^{3}\times 2\right)\times N$

N = The number of genes in the screening library, H = The number of genes a screen should uncover under ideal conditions, R_FN _= Fraction of reagents of H that fail. R_FP _= Fraction of reagents of N that are false positives. Rule "A": Only one reagent targeting a gene need to be a "hit" for the gene to be called a "hit". Rule "B": All reagents targeting a gene need to be a "hit". Rule "C": More than half of reagents must score as a "hit".

**Figure 6:**
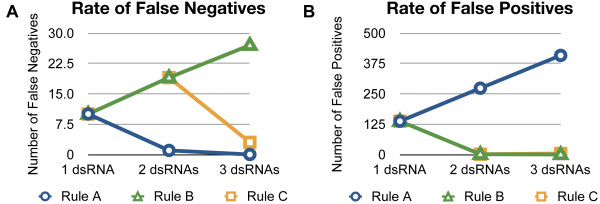
**Number of False Negatives and False Positives under hypothetical screening scenarios**. We assume a false positive rate of 1% and a false negative rate of 10%, a scenario of 100 "true hits" in the library, and a library targeting 13,735 protein-encoding genes. (A) The predicted number of false negatives with 1, 2, or 3 dsRNAs per gene under 3 different rules for interpreting ambiguous cases. (B) The predicted number of false positives with 1, 2, or 3 dsRNAs per gene under 3 different rules for interpreting ambiguous cases.

To illustrate the model, we chose as an example three hypothetical *Drosophila *genome-wide dsRNA libraries with false negative and false positive rates of 10% and 1% respectively (Figure 6). The model shows that the strategy used to disambiguate results from multiple reagents is critical when interpreting results from a library with more than one independently tested reagent per gene. In a hypothetical library with three reagents per gene, a lenient interpretation (requiring one or more of three reagents to score) results in few false negatives but an extremely high number of false positives in the outcome of a screen (Table 3 and Figure 6, Rule A). In this scenario, the presence of multiple reagents per gene virtually eliminates false negatives but at the cost of a high number of false positives as illustrated by our K/P JAK/STAT screen which would have a 62% final false positive rate (in terms of the percentage of hits) if interpreted this way. A stringent disambiguation (requiring all three reagents to score) results in few false positives but a high number of false negatives (Tables 3 and Figure 6, Rule B).

A third possible strategy for libraries with three reagents per gene (Tables 3 and Figure 6, Rule C) requires two out of three RNAi reagents to score. This disambiguation method achieved a balance of false negatives and false positives, resulting in low numbers of each relative to what would be achieved by screening a single dsRNA per gene. Thus, adding additional reagents per gene can greatly reduce false negative rates in screens but can also greatly increase the number of false positives in the absence of careful disambiguation.

For *Drosophila *cell-based RNAi screens, a library with three dsRNAs per gene, wherein discrepancies are disambiguated by requiring two of three dsRNAs to score, achieves a good balance between false negatives and false positives. For RNAi reagents with significantly different reagent-level false positive and false negatives rates, a different number of reagents with a different disambiguation strategy may be more appropriate. Indeed, several groups have proposed using four or more siRNAs per gene in mammalian siRNA screens [4,31]. Moreover, our model and disambiguation strategy is based on a simple binary interpretation of hits, but other more quantitative approaches have been proposed that do not require a screener to designate individual reagents as hits or non-hits. A recently described approach for disambiguating image-based RNAi screens, quantitative multiparametric image analysis (QMPIA), can be applied to complex screens with a very large number of read-outs [29]. A more broadly applicable quantitative disambiguation approach, the redundant siRNA activity (RSA) method [31], requires only one read-out per RNAi experiment. Regardless of the disambiguation approach used, screeners must carefully interpret results obtained with multiple reagents per gene in order to reduce false negative results without increasing the number of false positive results to an unacceptably high level.

## Conclusions

RNAi reagents that target the same gene do not always yield consistent results. Some of these inconsistencies can be explained by false positives and off-target effects, but some RNAi reagents are weak or ineffective and cause false negative results. False positive results and off-target effects can be partially filtered by using cell-line transcriptome expression data, and we have presented a web-tool to enable *Drosophila *cell-based RNAi screeners to filter screen results. RNAi libraries with multiple reagents per gene enable a reduction in false positive and false negative outcomes so long as care is taken when disambiguating inconsistent results to prevent an unintentional increase in false positive or false negative results.

## Methods

### Construction of the RNAi sub-libraries

RNAi "sub-libraries" were constructed by selecting genes based on known and predicted function as determined by FlyBase [19] supplemented with curation of the lists by experts. For each gene, two to four dsRNAs were selected from existing libraries or designed *de novo *using SnapDragon [21]. SnapDragon is a dsRNA design tool that selects gene regions common to splice forms and avoids sequences known to cause OTE [10,11]. RNAi reagents were constructed based on previously described protocols [32], dsRNAs were normalized to a dilution of 50 ng/ul, and 5 ul of this was aliquoted into each well of 384-well plates.

### JAK/STAT screen

A kinases and phosphatases sub-library screen was performed as described previously with minor modifications [22]. Briefly, S2R+ cells were transfected with dsRNA and two reporter constructs (10xSTAT-fire fly luciferase and actin-renilla luciferase). Three days later, the JAK/STAT pathway was stimulated via the addition of S2NP cells transfected with a plasmid expressing the JAK/STAT pathway ligand Unpaired (actin-Unpaired/act-upd) [22]. JAK/STAT signaling activity was quantified by measuring firefly luciferase activity, as the expression of firefly luciferase is under the control of 10 repeats of a STAT binding sequence. We used ubiquitously expressed Renilla luciferase activity to normalize for transfection efficiency and cell viability. The normalized luciferase values were used to calculate Z-scores. A Z-score for a well is calculated using the formula: (x - μ)/σ where x is the value of the well, μ is the mean value across all wells of the plate, and σ is the standard deviation of the well values of the plate.

### Cluster analysis

RNAi screens performed using the DRSC "2.0" genome-wide library (i.e. the most updated genome-wide library) were selected for analysis. Raw data from these screens were normalized using a standard plate-based Z-score analysis. The screens included are diverse in terms of assay read-outs and the subject under investigation; they include image-based screen assays, fluorimeter and luminometer (i.e. plate-reader) assays and investigated topics such as cell signaling pathways, pathogen infection, ion transport, cell viability, cellular and sub-cellular morphology, and RNA processing.

The 243 genes that target ribosome and proteasome components were selected based on Gene Ontology annotations in FlyBase [19]. A complete list of the dsRNAs analyzed in the study, which correspond to this set of 243 genes, can be found in Supplementary Table S1. The screen results obtained with dsRNAs targeting these genes were clustered based on their Z-scores across the screens using Cluster 3.0 [33] using Pearson's correlation and average linkage hierarchical clustering.

### Transcriptome analysis

To characterize gene expression levels, we used deep sequencing data obtained by the modENCODE consortium and available online [34] for the BG3, Cl.8, Kc167, S2-DRSC and S2R+ cultured cell lines. The first four cell lines were sequenced by modENCODE using 37 nt paired-end reads on the Illumina GAIIx platform (GEO Accession GSE15596) [18]. In addition, we analyzed samples obtained from the S2R+ cell line that were sequenced with the same platform in a strand-specific manner using a combination of single and paired-end reads of different lengths (76 nt and 108 nt, respectively. The reads were aligned the genome (FlyBase release 5.22) using TopHat [35] with up to two mismatches allowed and a mapping limit of 40 potential locations. Cufflinks [36] was used to estimate the level of expression of the annotated protein-coding genes. An FPKM (Fragments per Kilobase of gene/transcript model per million fragments mapped) value of 1 was set as a threshold for expressed genes. The expression of any gene in each cell line can be searched using the DRSC Cell Lines Expression Levels web-tool [26].

## List of abbreviations

RNAi: RNA interference; HTS: High throughput screening; OTE: Off-target effect; dsRNA: double-stranded RNA; DRSC: *Drosophila *RNAi Screening Center; K/P: Kinase/Phosphatase RNAi sub-library; TRXN: Transcription factors RNAi sub-library; UBIQ: Ubiquitin-related genes RNAi sub-library; TM: Transmembrane proteins RNAi sub-library.

## Competing interests

The authors declare that they have no competing interests.

## Authors' contributions

MB performed statistical analysis of DRSC RNAi screens and clustered dsRNAs that target ribosome and proteasome components, performed statistical analysis of the K/P JAK/STAT screen and interpreted its results, created the model of RNAi libraries with multiple reagents per gene and drafted the manuscript. AAS analyzed the deep-sequencing data for five *Drosophila *cell-line transcriptomes. YK performed the K/P JAK/STAT RNAi screen. IF developed the web-tool to query *Drosophila *cell-line gene expression data. SEM runs the DRSC, participated in screen design, and helped draft the manuscript. NP conceived of the study and helped draft the manuscript. All authors read and approved the final manuscript.

## Supplementary Material

Additional data file 1 **Supplemental Table S1: **dsRNA membership of the proteasome complex cluster and the cytosolic ribosome. Each gene is categorized as Proteasome, Ribosome, or Mitochondrial Ribosome. In bold are the 45 and 111 dsRNAs that belong to the proteasome and cytosolic ribosome clusters highlighted in Figure 2B and Figure 2A, respectively. Click here for file

Additional data file 2 **Supplemental Table S2: **dsRNAs targeting the cytosolic ribosome and proteasome complex components that failed to appear in their respective clusters. Click here for file
